# Short-term career perspectives of young cardiologists in the Netherlands

**DOI:** 10.1007/s12471-017-0971-6

**Published:** 2017-02-24

**Authors:** J. C. Vis, C. J. Borleffs, B. Zwart, R. J. Nuis, R. W. C. Scherptong

**Affiliations:** 1 Department of Cardiology, Academic Medical Centre, Amsterdam, The Netherlands; 2 Department of Cardiology, Leiden University Medical Centre, Leiden, The Netherlands; 3 Department of Cardiology, St Antonius Hospital, Nieuwegein, The Netherlands; 4 Department of Cardiology, Erasmus Medical Centre Utrecht, Rotterdam, The Netherlands

**Keywords:** Career choice, Employment, Contracts

## Abstract

**Background:**

Since it was anticipated that the need for doctors would increase due to demographic changes, the number of positions for medical specialty training programs has increased from the year 2000 onwards. However, the number of permanent positions for young cardiologists did not follow that trend leading to concerns about future employment. Therefore, the aim of the current study was to assess short-term career perspectives of young cardiologists in the Netherlands.

**Methods:**

All cardiologists who ended their training between 1 January 2011 and 31 December 2014 were invited to fill in a questionnaire about their first employment status and were followed yearly until the participant had a permanent position. The timespan between the end of training and the moment of permanent employment was assessed. Furthermore, the association between professional profile and short-term career perspectives was investigated.

**Results:**

The observed unemployment was 1.6% and lasted less than a year in all cases. Of the participants, 77% started their career with a temporary contract; within four years this was 7%. Of young cardiologists, 46% started their career as a fellow and 24% as an attending physician. A total of 29% of male cardiologists started their career with a permanent contract as compared with 12% of females (*p* = 0.01). Within two years this difference was no longer observed.

**Conclusions:**

Unemployment is low among young cardiologists. Most cardiologists start their career with a temporary contract. The time to a permanent contract is slightly longer for female cardiologists as compared with males.

## Introduction

The Dutch government regulates the number of cardiologists and other medical specialists by controlling the number of positions in medical specialty training. In the past decade, it was anticipated that the need for doctors would increase due to demographic changes such as population ageing. Therefore, the Dutch government decided to gradually increase the number of positions for most medical specialty training programs from the year 2000 onwards [1]. However, partly due to cutbacks in care, cooperative agreements among hospitals and hospital mergers, the number of permanent positions for most medical specialties have not followed that trend so far. This resulted in concerns about unemployment among young medical specialists. In response, the Dutch Federation of Young Medical Specialists issued a survey to investigate the career perspectives of young medical specialists. They reported unemployment rates varying from 5 to 15% depending on the type of medical specialty [2]. A major drawback of this survey was the low response rate (27%). To get a better view on early career perspectives, the Netherlands Society of Cardiology initiated a survey among young cardiologists. The primary goal was to investigate the unemployment rate and the timespan to obtain a permanent position. The secondary goal was to explore the association of professional profiles and short-term career perspectives.

## Methods

Participants were identified by direct contact with the cardiology departments of all training centres. All cardiologists who finished their training between 1 January 2011 and 31 December 2014 were invited to fill in a non-anonymous questionnaire online. The first questionnaire was sent on 1 October 2012. Besides personal characteristics, the questionnaire contained questions about the professional profile of the participant: PhD degree, area of expertise, the type of teaching institution (academic vs. non-academic), preference to work part-time (without defining working hours). Finally, participants were asked about their current employment status: employed vs. unemployed, permanent vs. temporary position, type of temporary position (fellowship, attending physician, locum physician). If participants indicated they had a permanent position, they were excluded from follow-up. If participants indicated they were unemployed, or had a temporary contract, they were included for follow-up. This follow-up questionnaire was sent yearly and contained questions about the current employment status as described above. Follow-up ended when a participant obtained a permanent position. If cardiologists were unavailable, information provided by colleagues or ex-colleagues about employment status was also approved (indirectly received data).

### Statistical analysis

Descriptive statistics were used to describe participants’ characteristics and type of employment position. Differences between the two groups were analysed by an unpaired Student t test. A multivariate analysis was performed to investigate the association between the baseline variables and the achievement of a permanent contract. Those variables found to be significant by univariate analysis (*p* < 0.1) were entered in the multivariate model. Statistical analysis was performed with the SPSS software for Windows XP version 16.0.

## Results

From 1 January 2011 to 31 December 2014, 193 residents ended their training program. For four young cardiologists, no information could be obtained (2 from 2011, 1 from 2012 and 1 from 2014), thus 189 participants could be included (98%). Of these 189 young cardiologists, information could be obtained from competed questionnaires in 142 cases. Data for the remaining 47 cardiologists was received. Table 1 summarises the baseline characteristics.

**Table 1:** Baseline characteristics

	Overall	2011	2012	2013	2014
Number of participants	189	57	48	42	42
Male (%)	129 (68)	39 (68)	36 (75)	26 (62)	28 (67)
Academic teaching hospital (%)	116 (61)	40 (70)	25 (52)	25 (60)	26 (62)
Having a PhD (%)	78 (41)	23 (40)	18 (38)	18 (43)	16 (38)
*Area of expertise*					
General cardiology	53	21	19	7	6
Imaging	37	10	9	9	9
Electrophysiology	28	9	6	5	8
Interventional cardiology	30	8	6	8	8
Intensive care	11	1	5	4	1
Grown-up congenital cardiology	4	3	0	0	1
Unknown	26	5	3	9	9

### First-year employment status

Three cardiologists were unemployed in the first year (all 2012), corresponding to an unemployment rate of 1.6%. In three cases, it was unclear whether or not the contract had a fixed term. All participants who were employed stated they worked as a cardiologist (4 participants worked abroad). Table 2 shows the first-year employment status and the types of positions. Overall, 23% of cardiologists received a permanent contract within 1 year. However, most participants started a fellowship (*n* = 87, 46%) or worked as an attending physician (*n* = 46, 24%). Since the first questionnaire was sent on 1 October 2012, participants from 2011 indicated they had a permanent position slightly more frequently (39%) as compared with the participants from 2012–2014 (15%, 21%, 14%, respectively). The participants who had a permanent position were most likely to work in an independent group practice (50%) or in an employed physician practice (36%).

**Table 2:** Employment status and job type

	Overall	2011	2012	2013	2014
Number of participants (*n*)	189	57	48	42	42
*Job status: temporary (n)*					
Fellowship	87	19	26	18	24
Attending physician	46	14	10	14	8
Locum (tenens) physician	2	1	0	0	1
Employed physician practice	4	1	2	0	1
Private out-patient clinic	1	0	0	0	1
*Job status: permanent (n)*					
Independent group practice	22	10	2	5	5
Employed physician practice	16	9	5	2	0
Not-specified	5	3	0	1	1
Private out-patient clinic	1	0	0	1	0
Job status: unemployed (*n*)	3	0	3	0	0
Job status: unknown (unemployed or temporary job)	2	0	0	1	1
*Job location*					
Academic hospital	61	16	21	13	11
General hospital (with CTSD, with RTP)	39	8	11	8	12
General hospital (no CTSD, with RTP)	39	15	6	9	9
General hospital (no CTSD, no RTP)	32	16	4	9	3
Private out-patient clinic	3	0	1	1	1
Not applicable (unemployed)	3	0	3	0	0
Working abroad	4	2	1	0	1
Unknown	8	0	1	2	5

*CTSD* cardiothoracic surgery department; *RTP* resident training program

A total of 163 participants (86%) indicated having an area of expertise. Table 1 shows a subdivision of the different areas of expertise. As expected, a large proportion (33%) indicated general cardiology as their area of expertise, followed by cardiovascular imaging (23%), interventional cardiology (18%) and electrophysiology (17%).

When analysing indirectly received data versus directly received data, no significant differences were found in gender distribution or proportion of permanent contracts.

### Professional profile and first-year career perspectives

Aspects of the professional profile of participants were assessed to analyse influence on first-year career perspectives.

#### PhD degree

Of the subjects, 41% indicated having a PhD degree and 30% of these had a permanent contract within the first year as compared with 18% of those without a PhD degree (*p* = 0.07). Subdivision into gender demonstrated that 23% of females with a PhD degree had a permanent contract as compared with 3% of females without a PhD degree (*p* = 0.046). Of the males with a PhD degree, 32% had a permanent contract within the first year as compared with 27% of males without a PhD degree (*p* = 0.69).

#### Type of teaching hospital

Of the participants, 61% were trained in an academic teaching hospital. Of these, 31% had a permanent contract within the first year as compared with 11% of participants trained in a non-academic hospital (*p* = 0.001). Subdivision by gender demonstrated that 21% of academically trained females have a permanent contract as compared with 0% of non-academically trained females (*p* = 0.02). Of academically trained males, 35% had a permanent position, as compared with 17% of non-academically trained males (*p* = 0.03).

#### Gender

In total, 68% of the cardiologists were male and 29% of them have a permanent contract within the first year as compared with 12% of the females (*p* = 0.01). Throughout the years, this tendency remained apparent. From the participants of 2011, 44% of males had a permanent contract compared with 28% of females. In 2012, this was 19% of males compared with 0% of females, in 2013 27% of males compared with 13% of females and in 2014 21% of males compared with 0% of females. Due to the small groups, statistical significance could not be calculated per year.

#### Preference to work part-time

To investigate if personal choices influence career perspectives we asked subjects if they would prefer to work part-time. Half of them responded: 65% of women and only 28% of men would prefer to work part-time (*p* = 0.001). Only 11 subjects with a permanent contract provided information about preference to work part-time.

#### Multivariate analysis

Multivariate analysis with the variables gender, PhD and type of teaching hospital was performed to analyse which factors were relevant for obtaining a permanent contract within the first year after training. This demonstrated that male gender (OR 4.4; 95% CI 4.5–13.6; *p* = 0.009) and being trained in an academic teaching hospital (OR 2.9; 95% CI 1.2–7.2; *p* = 0.022) were independent predictors of having a permanent contract within the first year after training.

#### Time to permanent position

**Fig. 1:**
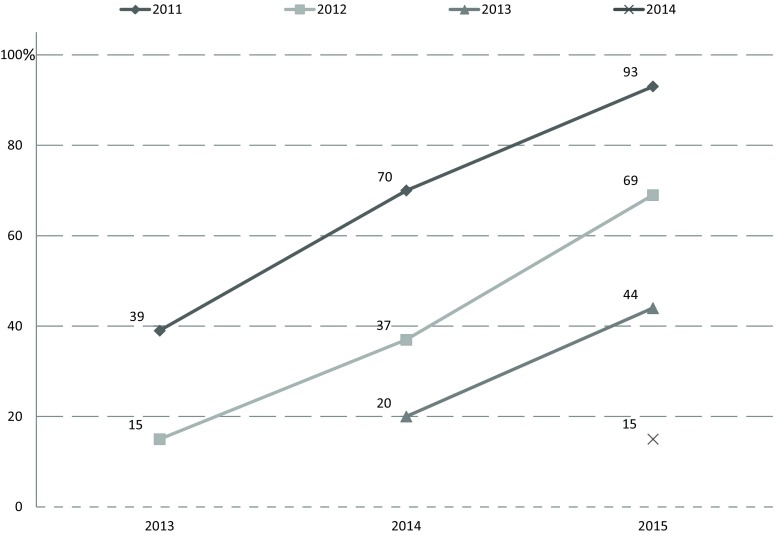
Time to permanent position. Each colour corresponds to a group of participants who ended their cardiology training within the same year. The numbers and dots indicate the percentage of participants that have a permanent position. The X‑axis denotes the year in which the (follow-up) questionnaire was sent. Thus, the individual lines indicate the evolution of the percentage of participants who have a permanent position

Follow-up (2011, 2012, 2013) was performed when participants did not indicate a permanent contract on the baseline questionnaire. Follow-up information was available from all participants of 2011 and 2013 and from 98% of 2012. Fig. 1 shows the evolution of the number of participants who obtained a permanent contract from the year of registration as a cardiologist, onwards. Of the cardiologists of 2011, 39% had obtained a permanent position in 2013. This increased to 93% 4 years after the end of training. Similar patterns were observed in the years 2012 and 2013.

The three unemployed cardiologists at baseline were all employed at follow-up (2 had a permanent contract and 1 a temporary position). The effect of gender on the time to obtain a permanent position was investigated as well. Of the group of participants who ended their training in 2011, 2012 or 2013, 69% were male. At first follow-up, 60% of males indicated they had a permanent position as compared with 37% of females (*p* = 0.07). At second follow-up, 81% of males as compared with 76% of females indicated to have a permanent position (*p* = 0.6).

## Discussion

The aim of the current study was to investigate short-term career perspectives of young cardiologists. The main findings were: (1) the unemployment rate was low (1.6%) and unemployment was of short duration, (2) most young cardiologists start their career as a fellow or as an attending physician, (3) the period of time between the end of cardiology training and the first permanent contract is slightly longer for females compared with males, (4) a fairly large proportion of young cardiologists have a permanent position within 4 years after the end of training.

To our knowledge, this is the first study on short-term career perspectives of young cardiologists in the Netherlands. As compared with the general population, in which the reported unemployment rate was 5.1% in January 2016, reported unemployment among young cardiologists is lower [3]. In the report of the Dutch Federation of Young Medical Specialists, the unemployment rate among young cardiologists varied between 5–8% [2]. However, the inclusion rate of that survey was lower than the current study (27% vs. 97%), which may lead to selection bias. Furthermore, no follow-up was performed to investigate the period of unemployment.

Most cardiologists start off their careers with a temporary position (77%) and less than 10% have a temporary contract after four years. Several factors may play a role in this observation. First, since subspecialties in cardiology are becoming increasingly important, a significant proportion of young cardiologists do a fellowship directly at the end of cardiology training. And, most of these fellowships are linked to a temporary contract. Second, the number of registered cardiologists has increased by 24% since 2010,[3] while at the same time, large hospital mergers and organisational changes took place on a national level in the medical specialist care sector [4]. The latter may have directed practices towards offering temporary positions in order to ensure organisational flexibility. Third, young cardiologists may also deliberately choose a temporary position to start their career with. It could be questioned if the observations of the current study could have an effect on the number of cardiologists to be trained. Since it takes several years to find a permanent position, one could argue that the number should be reduced. However, this number should mainly be adjusted to the amount of patients demanding cardiovascular care. Since the number of patients has sharply increased in past years and prognoses show that this will continue to increase, it seems reasonable that the number of cardiologists is in agreement with this trend [5, 6]. Furthermore, within their human resource policy practices should be balanced, on the one hand offering temporary contracts which increases organisational flexibility and, on the other hand, offering permanent contracts which ensures the career perspectives of young cardiologists. We do believe that the high number of temporary positions is undesirable, especially when this results in a sequence of multiple temporary positions for a cardiologist. However, our study did not show a significant increase in temporary positions during short-term follow-up. Furthermore, we do not know if the number of temporary contracts was lower in the past and we did not investigate the conditions of the temporary contracts and satisfaction level of temporary position holders.

Interestingly, this study shows a gender difference in career perspectives among cardiologists. The time between the end of training and getting a permanent contract is longer for females as compared with males. It could be hypothesised that choices in private life, such as family planning, may have an impact on career development. This may lead to longer fellowship programs and, thus, a longer period with a temporary contract. The higher number of women who preferred to work part-time may lead to an over-representation of women in temporary jobs as permanent jobs may require full time employees. Our data do not provide a clear answer to explain this observed gender difference in the first year. However, the gender difference was only of short duration as the rate of permanent positions was at follow-up.

In this study, the type of training centre appeared to be an independent variable for a permanent job. The national training program is similar across all centres so confounding factors, which we have not included in our study, play a role. Presumably the professional profile is different. However it was out of the scope of this study to investigate the influence of the professional profile on type of contract.

### Limitations

There are several limitations in this study. First, a short questionnaire was used to ensure a high inclusion rate. As a consequence, detailed information about participant characteristics and contractual conditions are lacking which hampers interpretation of the observations. Second, in an important number of cases, information was obtained from colleagues or ex-colleagues. However, since the study was focused on objective rather than subjective information, this probably has a limited influence on the study results.

## Conclusions

Unemployment is low among young cardiologists and of short duration. A large majority of young cardiologists start off their careers with a temporary contract. However, most cardiologists have a permanent position within four years of completing their cardiology training. The time to a permanent contract is slightly longer for female cardiologists as compared with males.
